# Leukocyte telomere length and its association with mammographic density and proliferative diagnosis among women undergoing diagnostic image-guided breast biopsy

**DOI:** 10.1186/s12885-015-1860-2

**Published:** 2015-10-30

**Authors:** Clara Bodelon, Christopher M. Heaphy, Alan K. Meeker, Berta Geller, Pamela M. Vacek, Donald L. Weaver, Rachael E. Chicoine, John A. Shepherd, Amir Pasha Mahmoudzadeh, Deesha A. Patel, Louise A. Brinton, Mark E. Sherman, Gretchen L. Gierach

**Affiliations:** 1 Hormonal and Reproductive Epidemiology Branch, Division of Cancer Epidemiology and Genetics, National Cancer Institute, Rockville, MD USA; 2 Department of Pathology, John Hopkins University School of Medicine, Baltimore, MD USA; 3 Departments of Pathology, Oncology and Urology, John Hopkins University School of Medicine, Baltimore, MD USA; 4 Department of Health Promotion Research, University of Vermont College of Medicine and Vermont Cancer Center, Burlington, VT USA; 5 Department of Biostatistics, University of Vermont College of Medicine and Vermont Cancer Center, Burlington, VT USA; 6 Department of Pathology, University of Vermont College of Medicine and Vermont Cancer Center, Burlington, VT USA; 7 Office of Health Promotion Research, University of Vermont College of Medicine and Vermont Cancer Center, Burlington, VT USA; 8 Department of Radiology and Biomedical Imaging, University of California, San Francisco, San Francisco, CA USA; 9 Breast and Gynecologic Cancer Research Group, Division of Cancer Prevention, National Cancer Institute, Rockville, MD USA; 10 Division of Cancer Epidemiology and Genetics, 9609 Medical Center Dr., Rm 7-E236, Bethesda, MD 20892 USA

**Keywords:** Telomere, Mammographic density, Breast pathology, Hyperplasia, Breast diseases, Breast neoplasms

## Abstract

**Background:**

Elevated mammographic density (MD) is a strong breast cancer risk factor but the mechanisms underlying the association are poorly understood. High MD and breast cancer risk may reflect cumulative exposures to factors that promote epithelial cell division. One marker of cellular replicative history is telomere length, but its association with MD is unknown. We investigated the relation of telomere length, a marker of cellular replicative history, with MD and biopsy diagnosis.

**Methods:**

One hundred and ninety-five women, ages 40–65, were clinically referred for image-guided breast biopsies at an academic facility in Vermont. Relative peripheral blood leukocyte telomere length (LTL) was measured using quantitative polymerase chain reaction. MD volume was quantified in cranio-caudal views of the breast contralateral to the primary diagnosis in digital mammograms using a breast density phantom, while MD area (cm^2^) was measured using thresholding software. Associations between log-transformed LTL and continuous MD measurements (volume and area) were evaluated using linear regression models adjusted for age and body mass index. Analyses were stratified by biopsy diagnosis: proliferative (hyperplasia, in-situ or invasive carcinoma) or non-proliferative (benign or other non-proliferative benign diagnoses).

**Results:**

Mean relative LTL in women with proliferative disease (*n* = 141) was 1.6 (SD = 0.9) vs. 1.2 (SD = 0.6) in those with non-proliferative diagnoses (*n* = 54) (*P* = 0.002). Mean percent MD volume did not differ by diagnosis (*P* = 0.69). LTL was not associated with MD in women with proliferative (*P* = 0.89) or non-proliferative (*P* = 0.48) diagnoses. However, LTL was associated with a significant increased risk of proliferative diagnosis (adjusted OR = 2.46, 95 % CI: 1.47, 4.42).

**Conclusions:**

Our analysis of LTL did not find an association with MD. However, our findings suggest that LTL may be a marker of risk for proliferative pathology among women referred for biopsy based on breast imaging.

## Background

Mammographic density (MD) is a radiological reflection of the fibroglandular content of the breast, which histologically corresponds to both increased epithelium and stroma [1]. Epidemiologic investigations have established that increased MD is a strong breast cancer risk factor [2], but the mechanisms that mediate the underlying risk are poorly understood [1].

Both environmental and biologic factors are thought to be responsible for the variations in breast tissue composition that are reflected in inter-individual differences in the extent of MD [3]. Factors associated with lower MD include increasing age, elevated body mass index (BMI) [3], and tamoxifen use [4], whereas nulliparity, later age at first birth, premenopausal status [5], menopausal hormone therapy use [6], and family history of breast cancer [7] are related to higher MD. Epidemiological factors associated with higher MD suggest that MD is related to cumulative exposures to hormones, growth factors or other factors that promote epithelial cell proliferation [3, 8]. However, biopsies of women with high MD vary with regard to severity of disease and epithelial content, and most women with high MD do not develop cancer. Accordingly, identifying which women with high MD harbor proliferative lesions that are associated with increased breast cancer risk is important. In contrast to markers that provide only a snapshot in time, telomere length captures replicative history, and therefore might reveal an underlying relationship with MD, another cumulative marker of risk.

Telomeres are nucleoprotein structures composed of repetitive DNA sequences (TTAGGG) and the shelterin protein complex. They cap the ends of chromosomes and help maintain genetic stability. TTAGGG repeats are lost during cell division, shortening telomeric DNA. When the telomeric DNA reaches a critical length, cells may undergo senescence, apoptosis or, if tumor suppressive mechanisms are abrogated, neoplastic transformation. Shorter telomeres have been observed in breast epithelial tumor cells compared with adjacent non-malignant tissue [9], with the shortest telomeres associated with the most aggressive subtypes of breast cancer [10]. In surrogate tissues (e.g., blood cells), associations between telomere length, breast cancer risk factors [11–16] and breast cancer risk [17–28] have been inconsistent.

As with MD, it has been suggested that shortening of telomeres could be a consequence of exposures that drive cell proliferation [29, 30]. We hypothesized that relative leukocyte telomere length (LTL), which may reflect cumulative exposures that promote cell division, may be related to MD. Therefore, we investigated the relationship between LTL and volume and area MD measures in a cross-sectional study of women referred for image-guided breast biopsy. Telomere shortening has been found to be involved in the early stages of breast carcinogenesis [29, 31], and therefore may be an indicator of subsequent malignant transformation. We also explored associations between relative LTL and proliferative versus non-proliferative biopsy diagnoses.

## Methods

### Study population

The National Cancer Institute (NCI) Breast Radiology Evaluation and Study of Tissues (BREAST) Stamp Project is a cross-sectional molecular epidemiologic study of mammographic density undertaken at the University of Vermont College of Medicine and its affiliated academic medical center, Fletcher Allen Health Care (FAHC). The study design and methodology have been described previously [32]. Briefly, 465 women who were referred for a diagnostic image-guided breast biopsy were enrolled between October 2007 and June 2010. Eligible women were 40–65 years of age, had not had breast cancer or received any cancer treatment, had not undergone breast surgery within the preceding year, did not have breast implants, were not taking breast cancer chemoprevention and were scheduled to have an image-guided breast biopsy.

The study was approved by the NCI Special Studies Institutional Review Board (IRB) and the University of Vermont IRB. Participants provided written informed consent to be part of the study and completed a standard health history questionnaireA research coordinator administered a telephone interview to collect additional health information. On the day of the breast biopsy, a research coordinator measured participants’ height and weight, and participants were asked to donate a blood sample. The informed consent included providing access to medical records and mammographic images and to breast pathology specimens not needed for clinical care. Compensation of $50 was provided to participants who opted to donate blood (processed and frozen as serum and blood clot) and/or mouthwash samples (processed and frozen as buccal cells).

### Assessment of pathologic diagnosis

Breast biopsy and surgical pathology reports were reviewed for all study participants. For the purposes of this analysis, diagnoses were classified as *non-proliferative* (i.e., benign; normal lobules or ducts defined as sclerotic/atrophied; non-proliferative fibrocystic change; other discrete non-proliferative benign breast diagnoses) or *proliferative*, including both atypical and neoplastic entities (i.e., ductal or lobular hyperplasia; sclerosing adenosis; in-situ carcinoma; invasive carcinoma). Information about biopsy type and laterality was abstracted from pathology reports.

### Assessment of mammographic density

Mammograms were acquired on one of six full field digital mammography systems at FAHC. Raw images were encrypted and transferred to the University of California at San Francisco for quantitative volume and area density assessment. This analysis was restricted to pre-biopsy cranio-caudal views of the contralateral breast. For women who underwent bilateral breast biopsies, the breast contralateral to the primary pathologic diagnosis was selected for analysis. If more than one mammogram was available, then the mammogram taken closest in time prior to the breast biopsy date was selected.

Breast density was quantified as an absolute fibroglandular tissue volume (cm^3^) and percent fibroglandular tissue volume using Single X-ray Absorptiometry (SXA), as described previously [33]. An SXA breast density phantom was affixed to the top of the compression paddle and included in the X-ray field during mammography examinations. Mammographic grayscale values were compared to the values of the SXA phantom. Previous estimates of reproducibility for the SXA test phantoms demonstrated a repeatability standard deviation of 2 %, with a ±2 % accuracy for the entire thickness and density ranges [33]. Area measures of density were estimated as described previously [34], using interactive, customized computer-assisted thresholding software comparable to other validated methods [35]. One trained experienced reader [34] measured absolute dense area (cm^2^) by setting a pixel threshold for dense tissue on the images. Percentage mammographic density was calculated by dividing the absolute dense breast area by the total breast area and multiplying by 100. For both area and volume density measures, distributions of density measures were examined and images with extreme values were reviewed visually for validation.

### Assessment of relative leukocyte telomere length

Whole blood samples were collected using standard techniques, allowed to clot for 30 min, and processed at the FAHC General Clinical Research Center. Samples were centrifuged at 3000 rpm for 15 min, and the serum and clot fractions were frozen at −80 °C until shipment to SeraCare Life Sciences (Gaithersburg, MD), where they were stored in liquid nitrogen. Leukocyte DNA was isolated from blood clots at SeraCare using phenol chloroform extraction methods and quantified at the Cancer Genomics Research Laboratory (Leidos Builmedical Research, Inc., Frederick, MD) with the QuantiFluor® dsDNA System (Promega) according to the manufacturer’s instructions. DNA in 500 ng aliquots was sent to Johns Hopkins University School of Medicine, where quantitative polymerase chain reaction (qPCR) was used to estimate the ratio of telomeric DNA to that of a single copy gene (β-globin) as previously described [36], with the following modifications. Briefly, to remove potential residual PCR inhibitors, leukocyte DNA was re-purified using a DNeasy Blood and Tissue column (Qiagen) and 4 ng of genomic DNA was used in a 25 μl volume for either the telomere or β-globin reactions; each sample was run in triplicate. The telomere reaction mixture consisted of 1× PCR buffer, 1.5 mM MgCl_2_, 100,000 fold dilution of SyberGreen, 200 nM dNTP mix, 1 % DMSO, 100 nM forward telomere primer (CGGTTTGTTTGGGTTTGGGTTTGGGTTTGGGTTTGGGTT), 900 nM reverse telomere primer (GGCTGGCCTTACCCTTACCCTTACCCTTACCCTTACCCT), and 0.8 U of Platinum Taq polymerase. The reaction proceeded for one cycle at 95 °C for 5 min, followed by 35 cycles at 95 °C for 15 s, and 54 °C for 30 s. The β-globin reaction mixture consisted of 1× PCR buffer, 2.5 mM MgCl_2_, 100,000 fold dilution of SyberGreen, 200 nM dNTP mix, 2 % DMSO, 300 nM forward β-globin primer (CACATGGCAAGAAGGTGCTGA), 700 nM reverse β-globin primer (ACAGTGCAGTTCACTCAG CTG), and 0.5 U of Platinum Taq polymerase. The β-globin reaction proceeded for one cycle at 95 °C for 5 min, followed by 35 cycles at 95 °C for 30 s, 58 °C for 30 s, and 72 °C for 45 s. Each 96-well plate contained a no template negative control and two separate 5-point standard curves ranging from 0.024 to 15 ng using leukocyte DNA. These standard curves allowed the PCR efficiency to be determined for each experimental run. Each of the 10 plates also included three samples isolated from a series of cell lines with known telomere lengths, ranging from 3 to 15 Kb, as determined by terminal restriction fragment analysis. Inclusion of these samples provided an additional quality control check. The coefficient of variation (CV) for this cell line series ranged from 1.3 to 6.8 % across plates. Samples were re-run if the CV of either the telomere or the β-globin reaction was equal or greater than 5 % or either the telomere or the β-globin values fell outside the range of the standard curve. The maximum CVs were 4.8 and 3.9 % for the β-globin and telomere reactions, respectively. The average β-globin threshold (C_t_) value and the telomere C_t_ value were calculated from the β-globin and the telomere triplicate reactions, respectively. For each sample, the telomere of the experimental sample to the single copy gene (T/S) ratio (−dC_t_) was calculated by subtracting the β-globin C_t_ value from the telomere C_t_ value. The relative T/S ratio (−ddC_t_) was determined by subtracting the -dC_t_ of the middle samples of the cell lines series from the -dC_t_ of each unknown sample. The relative T/S ratios (i.e., mean relative LTL) were used in the analysis.

### Analytic population

We restricted the study population to participants who had SXA volumetric MD measurements, donated blood and whose breast biopsies contained terminal duct lobular units (TDLUs) suitable for assessment of telomere lengths (analysis ongoing). Of the 465 participants who consented, 12 were not subsequently biopsied and were excluded; 338 (75 %) women donated blood and had clots with a volume ≥1.0 mL, of whom 212 also had breast tissues available for telomere length assessment. Twelve women were missing SXA density and were excluded. Characteristics of the remaining eligible 200 women as compared to and the rest of the participants in the BREAST Stamp Project were similar with the exception of BMI, which was lower in the women included in our analysis (data not shown). Of the 200 women whose DNA underwent qPCR for relative LTL assessment, two samples failed quality control on two separate runs and were excluded. In addition, three participants had a relative LTL that was larger than three standard deviations from the study population mean and were also excluded, resulting in a final analytic population of 195 women.

### Statistical analysis

Statistical differences in participant characteristics by biopsy diagnosis (proliferative vs. non-proliferative disease) were computed using the Wilcoxon rank-sum test for continuous measures and the *χ*^2^ test for categorical variables, except when values in cells where less than or equal to 5 in which case the Fisher’s exact test was used. The Spearman correlation coefficient was computed to examine the correlation between relative LTL with age stratified by pathological diagnosis. Logistic regression was used to compute the association between relative LTL (continuous) and risk of proliferative disease adjusting for age (continuous) and BMI (continuous), which are known to be strongly associated with relative LTL.

Relative LTL was transformed using the natural logarithm to improve normality. Multivariate linear regression was used to estimate the relationship between log-transformed relative LTL and participant characteristics adjusting for age (continuous) and BMI (continuous). Relative LTL was then back-transformed to the original scale and geometric means are presented. Similarly, multivariate linear regression was computed to examine the relationship between MD and relative LTL, adjusting for the potential confounders of age (continuous) and BMI (continuous). In sensitivity analyses, we additionally adjusted for age at first birth (nulliparous, <30, 30+ years) and menopausal hormone therapy use (premenopausal/postmenopausal ever/ postmenopausal never). Quantitative volume and area density measures were transformed by taking the square root to approximate a normal distribution. Relative LTL was modeled as either binary, by categorizing relative LTL at the median relative LTL levels in subjects with non-proliferative diagnoses, or as a log-transformed continuous variable. Adjusted means of mammographic density measures were back-transformed by squaring the results. *P*-values were two-sided and *P* ≤ 0.05 was considered statistically significant. All analyses were performed using the R software environment (version 3.0.2).

## Results

The majority of participants were diagnosed with proliferative disease (*n* = 141) and the remainder had a non-proliferative biopsy diagnosis (*n* = 54) (Table 1). Among women with a proliferative diagnosis, 110 had hyperplasia, 22 in-situ carcinoma and 9 invasive carcinoma. Among those with non-proliferative diagnoses, 42 had a benign diagnosis and 12 had other discrete non-proliferative diagnoses. Seven women with a proliferative diagnosis had bilateral biopsies. The diagnosis in the breast contralateral to the primary pathologic diagnosis for these women was benign for two of them, hyperplasia for four of them and in-situ carcinoma for one woman. None of the women with non-proliferative diagnoses had bilateral biopsies.

**Table 1:** Characteristics of women referred to an image-guided breast biopsy, by biopsy diagnosis

Characteristics	All women (*N* = 195)	Women with proliferative diagnosis^1^ (*N* = 141)	Women with non-proliferative diagnosis^2^ (*N* = 54)	*P*-value^§^
Age (years), mean (SD)	50.6 (6.4)	51.4 (6.4)	48.5 (6.2)	**0.005**
Age (years), *n* (%)				**0.040**
< 45	38 (19.5)	21 (14.9)	17 (31.5)	
45–49	51 (26.2)	36 (25.5)	15 (27.8)	
50–54	53 (27.2)	41 (29.1)	12 (22.2)	
≥ 55	53 (27.2)	43 (30.5)	10 (18.5)	
Race/Ethnicity, *n* (%)				1.00
White, non-Hispanic	184 (94.4)	*	*	
Non-White	11 (5.6)	*	*	
Education, *n* (%)				0.970
High school or less	29 (14.9)	21 (14.9)	8 (14.8)	
Some college	34 (17.4)	24 (17.0)	10 (18.5)	
College graduate or more	132 (67.7)	96 (68.1)	36 (66.7)	
Age at menarche (years), *n* (%)				0.476
≤ 12	75 (39.1)	57 (40.7)	18 (34.6)	
13	68 (35.4)	46 (32.9)	22 (42.3)	
≥ 14	49 (25.5)	37 (26.4)	12 (23.1)	
Parity, n (%)				0.280
Nulliparous	49 (25.1)	32 (22.7)	17 (31.5)	
Parous	146 (74.9)	109 (77.3)	37 (68.5)	
Age at first birth^¶^ (years), *n* (%)				0.639
< 30	105 (71.9)	80 (73.4)	25 (67.6)	
≥ 30	41 (28.1)	29 (26.6)	12 (32.4)	
Menopausal status, *n* (%)				0.103
Premenopausal	125 (64.1)	85 (60.3)	40 (74.1)	
Postmenopausal	70 (35.9)	56 (39.7)	14 (25.9)	
Age at menopause (years), *n* (%)				0.873
< 45	12 (19.7)	9 (18.8)	3 (23.1)	
45–49	17 (27.9)	13 (27.1)	4 (30.8)	
≥ 50	32 (52.5)	26 (54.2)	6 (46.2)	
Menopausal hormone therapy use^‡^, *n* (%)				**0.039**
Never	37 (52.9)	26 (46.4)	11 (78.6)	
Ever	33 (47.1)	30 (53.6)	3 (21.4)	
Body mass index (kg/m^2^), mean (SD)	25.9 (5.4)	25.8 (5.5)	25.9 (5.2)	0.649
Body mass index (kg/m^2^), *n* (%)				0.158
< 25.0	103 (52.8)	78 (55.3)	25 (46.3)	
25.0–29.9	53 (27.2)	33 (23.4)	20 (37.0)	
≥ 30	39 (20.0)	30 (21.3)	9 (16.7)	
First degree family history of breast cancer, *n* (%)				0.891
No	144 (73.8)	105 (74.5)	39 (72.2)	
Yes	51 (26.2)	36 (25.5)	15 (27.8)	
Primary pathologic diagnosis, *n* (%)				--
Benign	42 (21.5)	--	42 (77.8)	
Other discrete non-proliferative diagnosis	12 (6.2)	--	12 (22.2)	
Hyperplasia	110 (56.4)	110 (78.0)	--	
In-situ carcinoma	22 (11.3)	22 (15.6)	--	
Invasive carcinoma	9 (4.6)	9 (6.4)	--	
Mammographic density measure^¥^, mean (SD)				
% density (volume)	41.4 (21.5)	41.6 (20.9)	41.1 (23.0)	0.692
Dense volume (cm^3^)	193.5 (93.8)	195.1 (99.7)	189.2 (76.7)	0.992
Total breast volume (cm^3^)	603.8 (389.8)	607.8 (407.0)	593.3 (344.1)	0.776
% density (area)	30.6 (21.0)	30.0 (20.9)	32.1 (21.5)	0.539
Dense area (cm^2^)	35.9 (26.7)	35.3 (26.0)	37.6 (28.8)	0.886
Total breast area (cm^2^)	141.2 (69.3)	143.9 (72.7)	134.2 (59.3)	0.655
Relative leukocyte telomere length, mean (SD)				
Overall	1.5 (0.8)	1.6 (0.9)	1.2 (0.6)	**0.002**
Benign	1.2 (0.5)	--	1.2 (0.5)	
Other discrete non-proliferative diagnosis	1.3 (1.0)	--	1.3 (1.0)	
Hyperplasia	1.6 (0.8)	1.6 (0.8)	--	
In-situ carcinoma	1.8 (1.2)	1.8 (1.2)	--	
Invasive carcinoma	1.4 (0.7)	1.4 (0.7)	--	

*SD* Standard deviation

*For race, categories contained cell counts less than 5; data are not presented in order to maintain participant confidentiality

^1^Diagnosis of hyperplasia, in-situ or invasive carcinomas

^2^Diagnosis of benign or other discrete non-proliferative diagnosis

^§^*P*-value comparing proliferative and non-proliferative disease. *P*-value based on Wilcoxon rank-sum test for continuous variables and a *χ*^2^ test for categorical variables; for cells with values ≤5, we used the Fisher’s exact test

^¶^Restricted to parous women

^‡^Restricted to post-menopausal women

^¥^MD measures in breast contralateral to the primary pathologic diagnosis

The bolded numbers indicate statistical significance results at the 5 % level

Compared with women with non-proliferative diagnoses, women with proliferative diagnoses tended to be older (*P* = 0.005) and were more likely to have used menopausal hormone therapy (*P* = 0.039). MD measures did not differ between women with proliferative versus non-proliferative diagnoses. On average, relative LTL was similar among women with different categories of proliferative diagnoses (P-value comparing relative LTL in women with hyperplasia versus in-situ/invasive cancers = 0.71). However, women with proliferative disease had longer (unadjusted) mean relative LTL compared with those with non-proliferative diagnoses (Table 1; 1.6 (Standard deviation (SD) = 0.9) vs. 1.2 (0.6); *P* = 0.002). After adjustment for age and BMI, relative LTL was associated with a significant increased risk of proliferative disease (Odds Ratio (OR) = 2.46 per one unit increase of relative LTL, 95 % CI: 1.47, 4.42; Table 2). This relationship persisted when examining the association between relative LTL and in-situ/invasive cancer diagnoses versus non-proliferative disease (OR = 1.98 per one unit increase of relative LTL, 95 % CI: 1.07, 4.14). Similar odds ratios were observed after further adjustment for age at first birth and use of menopausal hormone therapy.

**Table 2:** Associations of relative leukocyte telomere length (LTL) with proliferative diagnoses

	Women with proliferative diagnosis^1^ (*N* = 141) Mean (SD)	Women with non-proliferative diagnosis^2^ (*N* = 54) Mean (SD)	OR (95 % CI)^*^	OR (95 % CI)^**^
Relative LTL	1.6 (0.9)	1.2 (0.6)	2.46 (1.47, 4.42)	2.41 (1.43, 4.38)
	Women with in situ or invasive cancer diagnosis (*N* = 31) Mean (SD)	Women with non-proliferative diagnosis^2^ (*N* = 54) Mean (SD)		
Relative LTL	1.7 (1.1)	1.2 (0.6)	1.98 (1.07, 4.14)	1.70 (0.86, 3.76)

*SD* Standard deviation

^1^Diagnosis of hyperplasia, in-situ or invasive carcinomas

^2^Diagnosis of benign or other discrete non-proliferative diagnosis

^*^Adjusted for age (continuous) and BMI (continuous)

^**^Adjusted for age (continuous), BMI (continuous), age at first birth (nulliparous/<30/≥30) and menopausal hormone therapy (premenopausal/postmenopausal ever/ postmenopausal never)

**Fig. 1:**
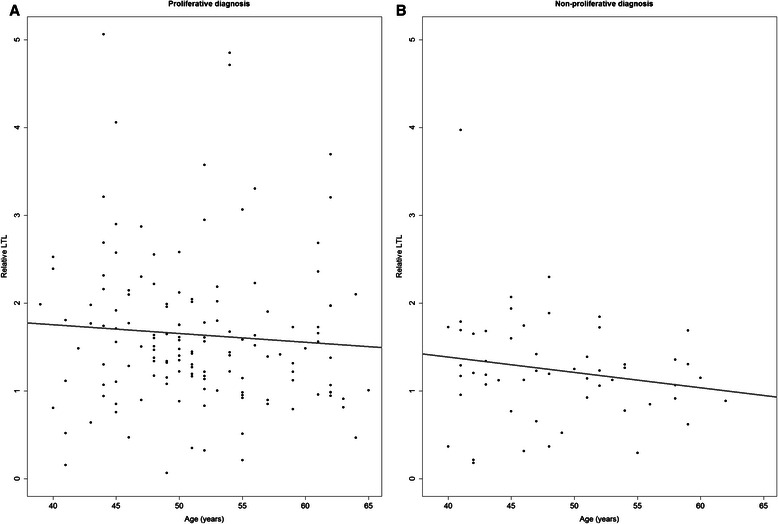
Relationship between relative leukocyte telomere length (LTL) and age by biopsy diagnosis. Individual LTL measurements (*black*) and linear regression fit to the individual measurements (*grey*). **a** Relationship between LTL and age in women with proliferative diagnosis (*N* = 141); Spearman’s correlation coefficient: −0.12 (*P* = 0.14). **b** Relationship between LTL and age in women with non-proliferative diagnosis (*N* = 54); Spearman’s correlation coefficient: −0.16 (*P* = 0.24)

Relative LTL was weakly and inversely correlated with age in women with proliferative and non-proliferative diagnosis (Fig. 1). After adjustment for age, longer relative LTL was associated with higher BMI, although this trend was statistical significant only in women with non-proliferative diagnosis (Table 3; *P* = 0.03). We did not find relative LTL to be associated with other risk factors such as age at menarche, parity, age at first birth, menopausal status, age at menopause or first degree family history of breast cancer. There was a suggested association between longer relative LTL and use of postmenopausal hormone therapy (*P* = 0.048).

**Table 3:** Age**-**adjusted geometric mean of relative leukocyte telomere length (LTL) in relation to participant characteristics

	All women (*N* = 195)		Proliferative diagnosis^1^ (*N* = 141)		Non-proliferative diagnosis^2^ (*N* = 54)		P-heterogeneity^¥^
Characteristics	Mean LTL^§^	(95 % CI) ^§^	Mean LTL^§^	(95 % CI) ^§^	Mean LTL^§^	(95 % CI) ^§^	
Age (years)							0.817
< 45	1.27	(1.05, 1.55)	1.47	(1.13, 1.91)	1.07	(0.80, 1.42)	
45–49	1.35	(1.14, 1.60)	1.47	(1.20, 1.80)	1.10	(0.81, 1.50)	
50–54	1.39	(1.17, 1.64)	1.46	(1.21, 1.77)	1.16	(0.82, 1.64)	
≥ 55	1.23	(1.04, 1.45)	1.31	(1.09, 1.58)	0.94	(0.65, 1.37)	
P-trend	0.774		0.423		0.745		
Body mass index (Kg/m^2^)							0.194
< 25.0	1.24	(1.10, 1.39)	1.35	(1.18, 1.55)	0.93	(0.73, 1.17)	
25.0–29.9	1.29	(1.09, 1.52)	1.44	(1.17, 1.78)	1.09	(0.83, 1.42)	
≥ 30	1.56	(1.29, 1.90)	1.57	(1.26, 1.96)	1.56	(1.05, 2.31)	
P-trend	0.058		0.248		**0.030**		
Age at menarche (years)							0.743
≤ 12	1.26	(1.09, 1.45)	1.33	(1.13, 1.57)	1.01	(0.74, 1.37)	
13	1.36	(1.17, 1.58)	1.53	(1.28, 1.83)	1.08	(0.83, 1.41)	
≥ 14	1.33	(1.11, 1.58)	1.43	(1.17, 1.75)	1.08	(0.76, 1.53)	
P-trend	0.613		0.530		0.787		
Parity							0.739
Parous	1.30	(1.17, 1.43)	1.39	(1.24, 1.55)	1.07	(0.88, 1.30)	
Nulliparous	1.35	(1.14, 1.61)	1.53	(1.23, 1.89)	1.08	(0.81, 1.44)	
Age at first birth (years)^*^							0.744
< 30	1.21	(1.08, 1.37)	1.30	(1.13, 1.50)	1.00	(0.79, 1.26)	
≥ 30	1.51	(1.24, 1.83)	1.66	(1.31, 2.10)	1.28	(0.90, 1.81)	
P-trend	0.066		0.084		0.246		
Menopausal status							0.361
Premenopausal	1.37	(1.19, 1.57)	1.40	(1.18, 1.66)	1.22	(0.98, 1.51)	
Postmenopausal	1.21	(0.98, 1.50)	1.45	(1.15, 1.82)	0.74	(0.47, 1.17)	
Age at menopause (years)							0.221
< 45	1.37	(1.03, 1.84)	1.79	(1.30, 2.46)	0.67	(0.39, 1.17)	
45–49	1.19	(0.94, 1.52)	1.28	(0.99, 1.66)	1.18	(0.68, 2.05)	
≥ 50	1.24	(1.04, 1.48)	1.31	(1.09, 1.58)	0.80	(0.52, 1.22)	
P-trend	0.655		0.166		0.654		
Menopausal hormone therapy use^‡^							0.461
Never	1.13	(0.95, 1.35)	1.26	(1.02, 1.57)	0.89	(0.60, 1.34)	
Ever	1.49	(1.22, 1.80)	1.50	(1.23, 1.84)	1.10	(0.60, 2.01)	
First degree family history of breast cancer							0.883
No	1.25	(1.13, 1.38)	1.35	(1.20, 1.51)	1.03	(0.85, 1.25)	
Yes	1.50	(1.27, 1.78)	1.64	(1.34, 2.00)	1.19	(0.87, 1.61)	

^1^Diagnosis of hyperplasia, in-situ cancer or invasive cancer

^2^Diagnosis of benign or other discrete non-proliferative diagnosis

^§^Based on linear regression with the log-transformed of the relative telomere length as outcome. Results are back-transformed. All characteristics were adjusted for age (continuous) and BMI (continuous), except age and BMI respectively

^¥^P-value based on a Wald test in the regression model corresponding to an interaction term of the proliferative disease variable times the corresponding variable (coded in continuous form)

^*^Restricted to parous women

^‡^Restricted to post-menopausal women

The bolded numbers indicate statistical significance results at the 5 % level

Linear regression models evaluating the relation between MD and relative LTL were adjusted for age and BMI, which were inversely associated with percent volumetric and area MD measures in the entire study population as well as in women with proliferative and non-proliferative diagnoses (data not shown). MD was not associated with relative LTL in our study population; this was true irrespective of the measure of MD (percent dense volume or area, absolute dense volume or area, and total breast volume or area) or biopsy diagnosis (Table 4). Similar results were obtained when modeling relative LTL as a continuous variable (data not shown).

**Table 4:** Age- and BMI-adjusted means of mammographic density (MD) in the breast contralateral to the primary pathologic diagnosis in relation to relative LTL

		All women (*N* = 195)		Proliferative diagnosis^1^ (*N* = 141)		Non-proliferative diagnosis^2^ (*N* = 54)	
Measure of MD	Binary LTL^¶^	Mean MD^§^	(95 % CI)^§^	Mean MD^§^	(95 % CI)^§^	Mean MD^§^	(95 % CI)^§^
% density (volume)	<1.20	37.81	(34.43, 41.34)	38.70	(34.47, 43.18)	36.48	(30.84, 42.59)
	≥1.20	39.22	(36.58, 41.94)	39.07	(36.15, 42.12)	39.57	(33.69, 45.93)
	P-trend	0.529		0.89		0.475	
Dense volume (cm^3^)	<1.20	182.28	(164.16, 201.35)	185.62	(162.26, 210.55)	175.14	(146.99, 205.75)
	≥1.20	185.24	(171.22, 199.81)	184.83	(168.77, 201.63)	188.37	(159.13, 220.08)
	P-trend	0.805		0.958		0.540	
Total breast volume (cm^3^)	<1.20	538.44	(487.35, 592.08)	538.35	(472.00, 609.05)	531.91	(452.82, 617.38)
	≥1.20	549.25	(509.65, 590.33)	548.35	(502.20, 596.52)	559.27	(478.09, 646.82)
	P-trend	0.749		0.814		0.649	
% density (area)	<1.20	26.98	(23.15, 31.11)	25.99	(21.21, 31.26)	28.84	(22.38, 36.12)
	≥1.20	26.25	(23.34, 29.33)	25.92	(22.61, 29.46)	27.27	(20.99, 34.36)
	P-trend	0.774		0.983		0.748	
Dense area (cm^2^)	<1.20	32.96	(27.71, 38.65)	32.22	(25.83, 39.31)	34.20	(24.87, 44.00)
	≥1.20	30.97	(27.06, 35.15)	30.79	(26.46, 35.45)	31.62	(22.68, 42.04)
	P-trend	0.566		0.729		0.718	
Total breast area (cm^2^)	<1.20	132.35	(122.72, 142.36)	135.33	(122.76, 148.52)	126.28	(111.12, 142.40)
	≥1.20	133.99	(126.56, 141.64)	135.66	(127.01, 144.60)	129.33	(113.98, 145.64)
	P-trend	0.796		0.967		0.788	

^1^Diagnosis of hyperplasia, in-situ cancer or invasive cancer

^2^Diagnosis of benign or other discrete non-proliferative diagnosis

^§^Based on a linear regression using square root of the density measure as outcome. Results are back-transformed. Adjusted for age (continuous) and BMI (continuous)

^¶^Telomere length was divided at the median levels in subjects with non

## Discussion

In this population of women ages 40–65 undergoing diagnostic image-guided biopsy, relative LTL was not associated with mammographic density. However, we observed a novel association between longer relative LTL and proliferative biopsy diagnoses.

Different measures of MD have advantages and limitations [37]; therefore, we explored the relation between relative LTL and MD using quantitative volume and area measures of MD. However, we found similar results irrespective of the MD measure used. As both MD and LTL are thought to, at least in part, reflect cellular proliferation in response to stimulation by hormones and growth factors, we had hypothesized that MD and relative mean LTL would be associated with one another. The observed null finding suggests that the biological determinants of MD and LTL differ. Notably, longer exposure to endogenous estrogen has been found to be related to longer telomeres [15], while support for a role of circulating estrogens in mammographic density is limited [38]. However, data suggest that elevated insulin growth factor-I (IGF-I) may be associated with both longer LTL [39] and with higher MD [3], whereas obesity is positively associated with IGF-I levels and negatively associated with MD. Interestingly, determinants of IGF family members have a strong, yet incompletely defined heritable component [40], like MD [7]. It is plausible that relative mean LTL is not a valid surrogate for telomere length in the breast. However, telomere length is highly heritable [41] and it is thought to have similar attrition rates in leukocytes and somatic tissues [42, 43].

The few studies examining associations between relative LTL and breast cancer risk factors in women without breast cancer have reported shorter telomeres with older age and higher BMI [11, 13, 44]; a relation between longer telomeres with increased number of reproductive years and age at menopause has been reported in some [12, 15], but not all [16], studies. Among the relatively narrow age range of women in our study, we observed a suggestive, albeit not statistically significant, inverse association between age and LTL. In contrast to prior findings [11, 13, 44], we found that relative LTL tended to increase with increasing BMI. While the reasons for this disparate finding are unclear, our inclusion criteria for this analysis may have led to an over-representation of leaner women; however, this association could also be due to chance as a consequence of the number of tests performed. Although we did not observe an association between relative LTL and age at menopause, our findings were suggestive of a positive association between LTL and ever use of menopausal hormone therapy, particularly among women with non-proliferative diagnoses. A previous study of postmenopausal women found that long-term users of hormone therapy had longer telomeres than never users [14], a finding which is also supported by in vitro experiments [45–47]. Results from these experimental studies suggest that there is an estrogen response element (ERE) located in the promoter region of the telomerase coding gene. In the presence of estrogen, the ERE induces transcriptional activation of telomerase [30, 46], which may result in the elongation of the telomeres.

In this population, we found that women with proliferative disease had longer relative LTL than women with non-proliferative disease. Women with different proliferative diagnoses (hyperplasia vs. in situ cancer vs. cancer) had similar mean LTL. Our study involved participants representing a range of breast biopsy diagnoses, which makes comparisons with prior studies that included a healthy control group challenging. Healthy control groups may include subjects with proliferative but non-cancer diagnoses, which are related to breast cancer risk. Nevertheless, our findings are consistent with some epidemiologic studies that have found that longer LTL is associated with increased breast cancer risk [17–21], but not with others that have observed inverse [24, 27, 28] or null [22, 23, 25, 26] associations. Although the association between longer relative LTL and breast cancer risk may seem counter-intuitive, telomerase, a reverse transcriptase that is able to synthesize telomeric DNA, may be upregulated in women with proliferative diagnoses in order to compensate for the shortening of telomeres related to their proliferative disease [48]. In addition, the upregulation of telomerase may allow cells to delay cell cycle arrest normally initiated by telomere loss, with additional potential for mutagenesis [20, 21].

A strength of our study was the use of quantitative, reliable density measures that have been validated with respect to breast cancer risk. Limitations include the relatively small sample size and narrower age range of participants than in most prior studies of LTL [49]. It is possible that an association between relative LTL and MD could be observed in a population with greater variation in mean LTL. Finally, we selected women whose breast tissues contained TDLUs suitable for future telomere length assessment in relation to TDLUs. It is known that presence of TDLUs is associated with elevated mammographic density [50] and therefore this select group of women may not be representative of the general population of women referred to breast biopsy. While it is possible that the method we used to select participants for this analysis may have obscured an association between relative LTL and MD, when we compared the distribution of MD measures for the women in our analysis with the rest of the study population, the age- and BMI-adjusted mean density measures, as well as the range of MD measures, between the two populations were similar.

## Conclusions

This is the first study to examine the relationship between MD and relative LTL, and importantly, in a population that may be at higher risk of breast cancer, for which biomarkers of risk may be critical. While relative LTL was not associated with MD, we found that longer relative LTL was associated with proliferative lesions among women undergoing diagnostic image-guided biopsy. This finding suggests that LTL may be a marker of risk for proliferative pathology among women with abnormal breast imaging prompting a biopsy. Additional studies with larger populations and broader age ranges are warranted.

## Abbreviations

BMI: Body mass index
CV: Coefficient of variation
ERE: Estrogen response element
FAHC: Fletcher Allen Health Care
LTL: Leukocyte telomere length
MD: Mammographic density
NCI: National Cancer Institute
qPCR: Quantitative polymerase chain reaction
SXA: Single X-ray Absorptiometry
TDLUs: Terminal duct lobular units
